# Supplementary material for: Lorentz-violating type-II Dirac fermions in transition metal dichalcogenide PtTe2
Source: Nat Commun. 2017 Aug 15;8:257. doi: 10.1038/s41467-017-00280-6 (PMC5557853; doi:10.1038/s41467-017-00280-6)
Supplement: Supplementary file 1 — Supplementary Information [file 41467_2017_280_MOESM1_ESM.pdf]

File name: Supplementary Information
Description: Supplementary Figures

File name: Peer Review File
Description:

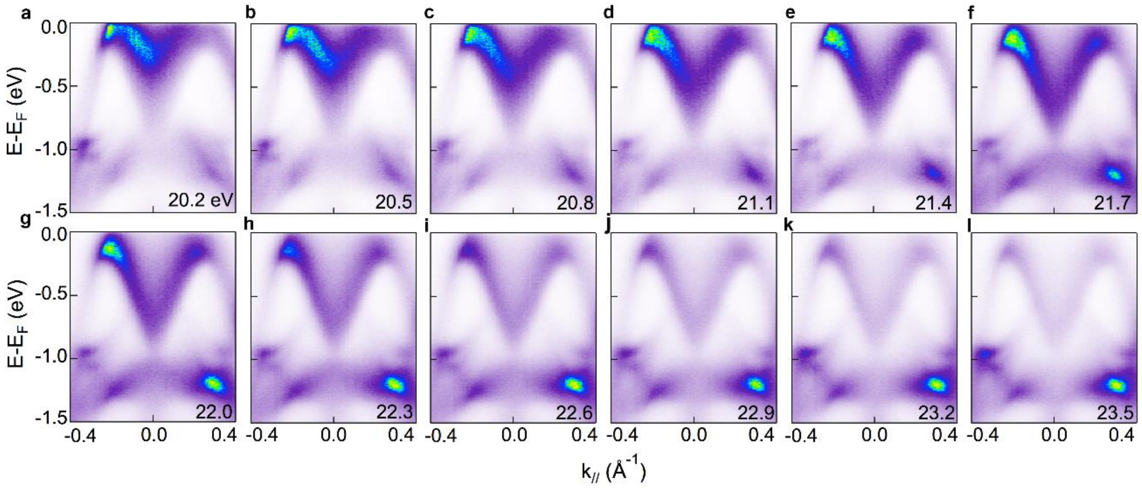

Supplementary Figure 1: Evolution of the dispersion along Γ-M with a finite k_z (photon energy) step. (a-l) Dispersions along Γ-M high symmetry direction at different k_z values from 0.28 to 0.41×2π/c with a fine photon energy step of 0.3 eV.

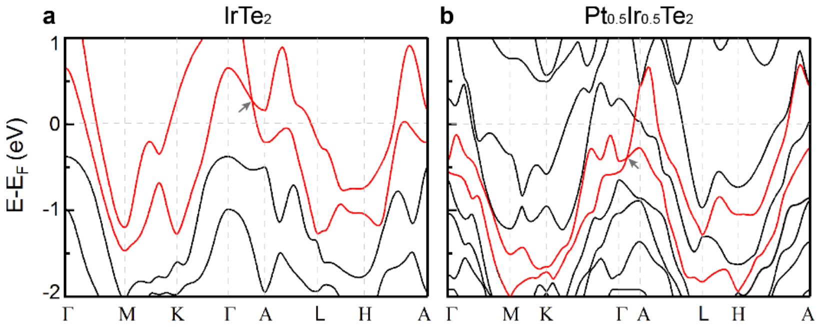

Supplementary Figure 2: Doping $PtTe_2$ with Ir to shift the Dirac node to the Fermi energy. (a) Calculated band dispersion of $IrTe_2$ along high symmetry directions, resolving the tilted Dirac node above the Fermi level. **(b)** Calculated band dispersion of $Ir_{0.5}Pt_{0.5}Te_2$ along high symmetry directions.
